# Experiencing and controlling time in everyday life with chronic widespread pain: a qualitative study

**DOI:** 10.1186/1471-2474-9-3

**Published:** 2008-01-11

**Authors:** Jane C Richardson, Bie Nio Ong, Julius Sim

**Affiliations:** 1 Primary Care Musculoskeletal Research Centre, Keele University, Keele, Staffs, UK

## Abstract

**Background:**

Chronic widespread pain (CWP) affects 10% of adults and often causes significant disability in everyday life. Research on time in chronic conditions has focused on biographical disruption and perceptions of past and future. However, more mundane aspects of time are also disrupted in a condition such as CWP, which is uncertain on a minute-to-minute, day-to-day basis, as well as in the longer term. The results presented here are part of a wider study, the aim of which was to explore how people with CWP experience and give meaning to their 'condition'. This article focuses on how mundane, repetitive and taken-for-granted aspects of everyday life are disrupted for people with CWP.

**Methods:**

Eight people aged 40–60 years living with CWP took part in multiple in-depth interviews, diaries and family interviews, exploring the meanings and interpretations of participants and individuals' experiences in a social context.

**Results:**

The findings illuminate the ways in which the experience of time is changed by CWP: carrying out the tasks of everyday life takes longer, routines are disrupted, and changes are needed in how time is managed. Some strategies for managing these tasks rely on ability to control one's time. However, this is not always possible and, for some, the experience of CWP becomes characterised by lack of such control.

**Conclusion:**

This study explored the concept of controllable time in the experience of CWP. Regaining control over time is an important element in coping with chronic pain, and helping patients to regain such control has potential as a target for health professionals involved in pain management.

## Background

Everyday life is characterised by a number of features, including a distinctive sense of time, in terms of repetition; habit, or taken-for-grantedness; and spatial ordering, focused, although not exclusively, on the home [1]. Living everyday life with chronic widespread pain is not simply about *experiencing *pain in a daily context, but is also about *managing *pain in the context of daily routines and activities, and, additionally, managing everyday routines and activities in the context of pain. If, as Bennett and Watson suggest, the "mundane, repetitive and taken-for-granted aspects of everyday life are of considerable significance in the life experiences of specific individuals and groups ([2], pxxiii)," then an exploration of these same aspects among people with chronic widespread pain will illuminate the ways in which their lives are altered by the condition.

Research on concepts of time in chronic illness has focused on biographical disruption – the disruption to existential time and perceptions of the future in life-threatening illnesses, and the alteration of perceptions of past, present and future [3-5]. Chronic illness, by definition, has a temporal nature [6], and these concepts are all relevant to conditions which are characterised partly by uncertainty, including chronic widespread pain [7,8]. However, more mundane aspects of time are also disrupted in a condition such as chronic widespread pain, which is uncertain on a minute-to-minute, day-to-day basis, as well as in the longer term.

Corbin draws attention to the importance of different types of time in health and illness, distinguishing between "clock time," "historical time," "biographical time," "perceived time," and "internal time [9]." Historical time (the development of one's body over time, including family influences and genetic factors) and biographical time (lifetime experiences which make up a self and which may be disrupted by illness) are both relevant to work on past, present and future in chronic illness. Clock time, perceived time and internal time are more relevant to the everyday lives of people with chronic widespread pain. In this article we focus on clock time and perceived time and introduce the notion of "controllable time" as an important aspect of the experience of living with chronic widespread pain. We use clock time to refer to time related to tasks or routines to be carried out at particular points, often determined externally; for example, work or taking of medicines. We use perceived time to refer to how a person feels about time in their everyday life, whether it is that something is taking a long time to complete, or that a task cannot be sustained for any length of time. Perceived time may be taken for granted in health, but not in illness.

The aim of the wider study on which this article is based was to explore how people with chronic widespread pain experience, understand and make meaning of their 'condition', and attempt to influence or exert control over their pain [7,8]. The aim of this article is to explore the concept of time in the everyday lives of people with chronic widespread pain.

The article is primarily a sociological analysis, but in the conclusion we will draw out some suggestions as to how our findings may inform clinical practice.

## Methods

Ethical approval for the whole study was obtained from North Staffordshire Local Research Ethics Committee.

### Participants

We selected participants from responders (who had agreed to further contact) to a previous community-based survey of adults carried out in 1995, designed to explore other health areas but including questions on musculoskeletal pain. Potential participants for the qualitative study were selected on the basis of having pain that was chronic (had lasted for at least three months) and widespread (occurring in the axial skeleton, including low back, above and below the waist and in contralateral limbs, these being the classification criteria for chronic widespread pain) [10]. The final sample of eight people therefore comprised people who would have experience of chronic widespread pain, and also reflect a range of experience related to age, length of time with pain, family circumstances and so on. They also had to be willing to take part in the full qualitative study. The four men and four women ranged in age from 40–60 years. All except one had stopped work due to their health. Table 1 provides details of the participants.

**Table 1:** details of participants

**Pseudonym**	**Age at interview**	**Family**	**Work**
**Duncan**	50	Wife; 1 child at home, 1 child away	Retired from police
**Eileen**	57	Single; no children	Retired from teaching
**Harry**	53	Wife; 1 adult child at home	Previously factory fitter/foreman and warehouse work
**Michael**	56	Wife; 1 child at home, 1 child at university	Previously miner and railway guard
**Natalie**	40	Husband; 1 child at home	Previous work, last as phlebotomist
**Sue**	46	Husband; 1 adult child with own children; 1 adult child at home	Part-time work in warehouse
**Trevor**	58	Divorced; 1 child (no contact)	Previous manual work
**Val**	54	Husband; 2 adult children at home	Previous manual work in pottery industry

### Approach

In this study we took an interpretative psychosocial approach, focusing on the meanings and interpretations of participants, who are considered as active agents in managing and responding to illness [11], and exploring individuals' experiences in a social context. We also drew on a narrative approach, in the sense of using the stories told by research participants to explore various aspects of chronic widespread pain in their everyday lives, but within the context of cautions raised over this approach [12,13], for example that the analysis of narrative forms or types – that is, the *way *in which people structure and tell their story – should not take precedence over the 'everyday and mundane dimensions of experience' [12].

### Data generation

We used a combination of data generation methods, centred around multiple in-depth interviews, which enable process or change (or indeed stability) to be highlighted, trust to be built [14], and a deeper understanding to be gained of 'the meaning which informants attribute to certain events' [15]. Each participant was interviewed by JCR on three occasions over a period of eight months (2001–2002): first, using a lifegrid; second, a follow-up to the lifegrid interview; and third, an interview based on an unstructured diary, which seven participants completed. JCR also interviewed five family members (four spouses and one daughter). All interviews took place in participants' homes.

**Figure 1:**
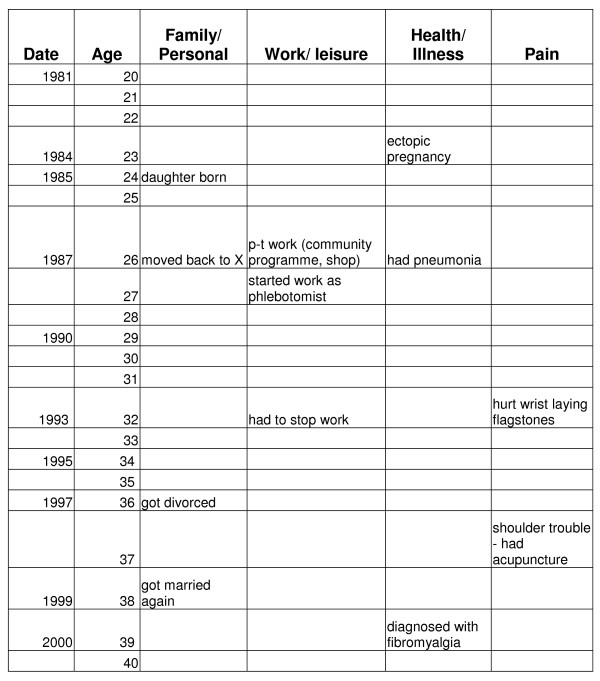
Extract from Natalie's lifegrid.

Lifegrids (see Figure 1) provide one way to obtain information on participants' life courses and enable the creation of a "diagrammatic chronology" of a participant's life [16,17]. In practical terms a lifegrid is a chart with rows showing years in a participant's life, labelled with the actual year and the age of the individual in that year, at five or ten year intervals. Different areas of a participant's life are represented in the columns of the lifegrid, for example family, work/leisure and health [16]. A lifegrid enables the illness experience to be incorporated into the life story and takes into account that illness does not just *impact *on the areas of a person's life, but is *experienced *there [18]. The flexible nature of the lifegrid, combined with the movement towards task completion, means that participants can set the pace of the interview, while the joint completion of the task creates a rapport between interviewer and participants, helping to build a foundation for the follow-up interviews. The follow-up questions focused on the completed lifegrid and asked participants to elaborate on particular areas. Lifegrid and follow-up interviews lasted between one and two hours.

Diaries are useful for revealing the mundane daily activity of dealing with bodies in pain, an important and under-researched area in the study of chronic illness and chronic pain [12,19,20], and diary interviews allow topics that arose in diaries to be explored further. The diaries were unstructured and were left with participants for three weeks. Emphasis was placed on including any information that participants thought relevant, with examples given including how pain affected daily routine, anything done to relieve the pain and anything that happened which affected the pain. Diary interviews lasted between forty minutes and an hour.

Family members were interviewed in order to understand the experience of chronic widespread pain within a wider social context and to explore how people construct their condition and identity within different circumstances and relationships [21]. These interviews were semi-structured, adapted to individual participants. One couple chose to take part in all their interviews together; one participant remained in the room while her husband was interviewed; the remaining interviews took place solely with the family member. Family member interviews lasted between thirty minutes and an hour.

The research was carried out in line with the British Sociological Association guidelines for conduct of ethical research [22]. Participants gave written informed consent for each interview, re-affirmed at the end of the interview. All tapes and diaries were recorded and transcribed fully and NVivo 1.3 [23] was used to manage the data. All names and details given here have been changed in order to preserve participants' anonymity.

### Data management and analysis

Analysis was a combination of the thematic and the narrative. We attempted to use analytical methods appropriate to the different material generated by participants, drawing on, and combining elements from, different systems of coding and analysis, in particular, Interpretative Phenomenological Analysis (IPA). This process involves identifying issues of interest in transcripts, which are categorised as themes, and then clustered into 'superordinate themes'. The 'master list' of themes is then used to inform the analysis of the other data [24]. IPA is cyclical in nature, allowing themes to be dropped, or the focus altered, depending on what emerges from the data and with the analysis of subsequent data and is also flexible, facilitating its adaptation to encompass data from different sources, including interviews and diaries. Initial analysis was carried out by JCR and discussed with BNO and JS, which informed the subsequent full analysis.

## Results and Discussion

The concept of time manifests itself in various ways throughout the everyday experience of living with chronic widespread pain. In this section we focus first on participants' descriptions of "typical days" and how they balance the uncertainty of their pain with routine; and second, on the concept of control, or lack of control, in the everyday lives of people living with chronic widespread pain.

### 1) Typical days: routine in everyday life with chronic widespread pain

Participants' accounts of "typical days" reveal the "minutiae that make up daily living [25]." However, these accounts also reveal a volatility that challenges the notion of typicality, a volatility acknowledged in the title of Charmaz's work on chronic illness ("Good days, bad days") [5]. Nevertheless, participants' descriptions of what they consider to be a typical day are useful in understanding how they manage the uncertainty of their chronic widespread pain, and it is these that we shall consider, before going on to explore how the concept of time manifests itself through the specific activities of everyday life.

Typical days for people with chronic widespread pain are shaped by a pre-set routine, by the level of pain on that day, or by a balancing of these two factors. For example, Harry's routine is shaped by walks with the dog – in the morning to the newsagents, then after lunch, and again after tea:

***Harry: ****I mean if me feet were that bad, but I still make myself go *[to the] *paper shop, I always, because that is part of the exercise. Otherwise if you sit in a chair, you are going to seize up. So no matter how much I am hurting, I still do the exercise, that is part of doing the exercise, of maintaining*-

***Carol (Wife)****: – just takes you longer*.

***Harry****: Yeah that is true. Because you sometimes wonder where I have been, don't you? Say, 'God, how long has it taken you this morning?'*

[Harry (male, 50s), Carol (50s) follow-up to lifegrid interview]

The way in which Harry manages the balance of his everyday life is shaped by his belief that his condition will "*burn itself out*" and leave his body in the state it is at that time. He believes it is important to not allow his body to "*seize up"*, thus the walks described in the extract form part of his overall exercise regimen. He emphasises that he always does his walk, even if he is in pain, although it may take him longer. So, although he attempts to limit the uncertainty in his day through forcing his body to maintain its routine, the uncertainty manifests itself in the time taken to complete this routine.

Natalie evaluates a day as "good" or "bad", based on her pain levels. This then shapes that day, in combination with her aim to get out of the house for some part of every day:

I wouldn't necessarily walk very far, but erm, if it is a really good day then I'd probably think about cleaning the bedrooms, you know, do some housework. But my husband and daughter are brilliant, they help, yer know, a lot. But if I am feeling good I think 'Right what can I do?'

[Natalie, 40s, lifegrid interview]

These words convey the idea of the strategy of doing what you can when you can, or of 'storing up' housework against an (unknown) time when it cannot be done, similar to the strategy of "just-in-casing" identified in previous research [26]. This also relates to the notion of perceived time: for Natalie, time free from pain cannot be taken for granted and therefore has to be used carefully through controlling the activities carried out.

The most frequently described scenario was a combination of pre-set routine, with alterations made according to evaluation of the pain level on a particular day:

*If I'm feeling all right in myself, I'm not having problems, with the two problems that I mentioned in the diary I would say that, I would treat each one on its own, how can I say, try and stick to set routine if I'm not feeling too bad in myself, but then when the problems start, I tend to go the opposite way, I don't stay to routine. I just do it whenever I can*.

[Trevor, 50s, diary interview]

This extract illustrates the delicate balance between daily routine and fluctuating levels of pain. Trevor's ability to control his routine to some extent is contrasted with the uncontrollable nature of the pain, which makes the routine impossible to maintain.

While pain levels on a particular day can influence the balance of activities on that day (the notion of good days and bad days), routine can also be influenced by various other factors, including external commitments. The family context may also influence routine: for example, a typical day for Natalie is determined by what shift her husband is working. Val's morning routine is determined by a balance between her need to have someone in the house while she has a shower or bath, and the routines of her family. Although participants in this study generally needed little help with personal care activities, for Val the unpredictable nature of her pain means that, although she is *able *to bathe alone, she only does so when one of her family is in the house, getting up at 5.30 in the morning in order to do so. Val's self-care activities are therefore structured around clock-time, in that a specific activity has to be carried out at a particular time. Val reports saving money in order to buy a bath seat, which will remove the need for the presence of a family member and thus give her more control over her time and activities, illustrating how the ability to control one's time is related to economic circumstances.

Normal family routines may also be disrupted by chronic widespread pain. Sheila describes the changes since her husband stopped work:

*Sometimes in the morning I think, 'Oh no,' because he used to go to work early. Sometimes when he gets up earlier than usual, I think, 'Oh no,' because we are all under one another's feet some how as we weren't before*.

[Sheila, 50s, wife of Michael]

This short description is indicative of the changes in Sheila's relationship with time and with her domestic "space", created by her husband's chronic widespread pain. The previous routine of the household, shaped by Michael leaving for work early, to meet the demands of clock time, is now changed, leading to a need for adjustment to the movement of bodies within the space of the household. Thus control over time, in the sense of an established routine, is also changed as a result of chronic widespread pain, although this change is due to a *loss *of activities related to clock time.

Charmaz suggests that good days and bad days are defined according to the intrusiveness of illness [5]. The participants in our study use their knowledge of their bodies to evaluate the level of pain and hence determine what activity can be carried out in that day. The notion of good days and bad days is thus inextricably bound up with accomplishment of activities. It is also clear that good days and bad days are linked to a sense of self and identity [5]. A good day allows one to carry out activities that are an important part of identity, whether these are gardening or housework. Similarly, Corbin [9] describes the way in which time in health allows people to carry out "activities associated with self-chosen roles (p259)."

The issue of control, over bodies and time, manifests itself in participants' descriptions of typical days. On a good day the body can be controlled in order to accomplish activities, and to store up time against a bad day, on which control, and routine, are disrupted. It has been suggested that (in the case of relapsing-remitting multiple sclerosis) the fluctuating nature of the symptoms creates the challenge of 'squeezing' activity into good days and preserving energy for bad days [27]. The uncertainty in the experience of chronic widespread pain may contribute to the similar tensions and striving for balance seen in participants' accounts of managing their everyday lives.

### 2) (Lack of) control over time and bodies in everyday life with chronic widespread pain

The concept of "controllable time" is implicit in Corbin's breakdown of different types of time in health and illness and is also, as we will show in this section, an important part of the everyday life of people with chronic widespread pain.

People with chronic widespread pain experience a lack of control over time through deployment of their bodies in managing the activities of everyday life, first through the increasing time it can take to complete tasks, and second, through an inability to sustain tasks for any length of time. Both of these thus require a change in how time (and bodies) are managed and perceived. In this section we present the accounts of the impact and experience of pain alongside participants' description of the strategies they use to manage this, mirroring the ways in which participants describe their experience [12].

Nearly all informants describe similar problems with the physical activities associated with personal care, in particular using a bath, illustrated here:

*Another thing we're in the process of doing at the moment is ripping the bath out *[...] *because nobody uses it and I have great difficulty climbing over, in and out of the shower. So we're going to *[...] *have a proper shower cubicle and tray so that'll make life a lot easier, a lot easier that will, much better. Other than that I think it's just a matter of now I think I'm used to it now, and I think, 'Hang on, you've got to do this and it's going to take you so long to do so you've got to take your time and you will have to take your time and do it*'.

[Duncan, 40s, follow-up to lifegrid interview].

Duncan uses the bath to illustrate his approach to everyday life problems, an approach that centres on time. He 'allows' himself time to complete tasks based on knowledge that it will take him "so long" to do, his words suggesting that he is permitting himself to take the necessary length of time. Illness cuts in to clock time, and alters perceived time because tasks take longer, as illustrated through his acknowledgement of the length of time it takes him to accomplish a task.

The other areas of everyday life in which pain reduces control over time and body are standing for any length of time, for example to iron or to prepare vegetables, and sustaining a physical task for any length of time, such as vacuuming or dusting. The main strategies participants report using for overcoming these problems are to control the relationship of the body to the physical task through sitting down to do a task that is normally done standing up; and to control the time taken to complete the task, through breaking the task up and completing small parts, interspersed with resting, expressed by Duncan as "*do a bit, sit down for a bit*." This strategy is also described in other studies of chronic illness and pain [27-29].

The strategy of "do a bit, sit down for a bit" could be applied to any task, but enacting it necessitates the ability to manage or control one's own time, and to manage the potential effects on identity. The description of household chores by Duncan and Becky in relation to time provides further illustration of this concept and its meaning for people with chronic widespread pain. Throughout Duncan's interviews he returns to the idea of how slowly he performs tasks in the house; for example, in this extract from his diary:

*Did a little housework, dusting etc. The only way I can do it is to do a little bit then sit down and rest, but again the rests seem to be getting longer. Still the house is clean and tidy, as you know I can't abide untidiness*.

[Duncan, 40s, diary]

Duncan manages the housework through his strategy of 'doing and resting' but is aware of the increasing time of the rest periods – a changing perception of the time available to him. However, he balances this against the knowledge that the house is clean, which is important to him. Thus, he rationalises the time taken in terms of the result, similarly describing how one of the consequences of his wife being on holiday from work is that "*the household chores, which takes ages to do, can be done so much more quickly*." For his wife, however, time takes on a different meaning, in that it highlights her husband's condition:

*Well I continued working full time. Duncan is the 'household engineer'. I think he gets frustrated because he can't do things as quickly as I can. He'll hoover *[vacuum] *a room, that is it for the day, whereas I hoover the whole house in half a day. I am very much aware of that fact. He does find it frustrating and I do tend to slow down on odd things I'm doing when I'm off for the day, thinking 'Well it would be rubbing it in a bit if I whiz through the house in half a day, whereas it would take him a week to do'. I am aware of that*.

[Becky, 40s, Duncan's wife]

This extract illustrates the contrasting relationship that this couple have with the physical environment, with clock time and with perceived time [9]. Becky's awareness of how much more quickly she can complete tasks leads her to manipulate, or control, time and the physical environment in order to reduce the salience of her husband's condition. She thus plays a collaborative role in maintaining his positive identity [30].

The case outlined above illustrates the importance of family help in completing household tasks and also how this highlights the issue of time and control over time. For example, restrictions are placed on when certain tasks can be completed, thus further disrupting the routines of everyday life:

***Int****: What about weekends? Do you sort of do things sort of differently?*

***Val****: No, just generally, I mean that is when the biggest part of the washing and ironing has to be done then. So erm*, [daughter] *has to sort of help me with that, you know*.

[Val, 50s, lifegrid interview]

For Val, the weekend is marked not by an *absence *of work, but by the *presence *of work, in the form of washing and ironing. Chronic widespread pain disrupts the rhythms of daily household tasks, reducing the control that sufferers have over time. Having to wait for someone to help means relinquishing control over time.

One solution to the lack of control over time due to reliance on other people to perform or help with household tasks is to find strategies to reduce this lack of control:

*I've got a piece of dust over here and I thought, 'I will get that thing *[mini-sweeper] *out when [interviewer] has gone later on this afternoon'. So I mean you're always on the look out for things make life a little bit easier for you, so it gives you that bit of – you haven't got to rely on somebody coming and doing that for you*.

[Val, 50s, diary interview]

Val reports noticing that things that need doing because she has a closer relationship with her everyday physical environment, and because she has the time to notice. Her use of a mini-sweeper not only reduces her reliance on other people but also leads to increased control over her environment and over her time. This supports the suggestion that a mechanical device is preferable to relying on a person, because of the issue of reciprocity, and as illustrated above, the connotations of dependence, and the issue of control [26]. Similarly, in a previous study some women did not ask for help with all tasks in order to retain control [29].

## Conclusion

Chronic widespread pain arises, is experienced, and is managed, in the realm of sufferers' everyday lives. In this article we have shown how the concept of time manifests itself in various ways in the everyday lives of those living with chronic widespread pain. We have also shown how people and their families attempt to control time in order to minimise the impact of the condition. Although the concept of controllable time underlies some of Corbin's elements of time in health and illness [9], our study suggests that the notion of controllable time can be identified more explicitly in the accounts of people with chronic widespread pain and those of their families as an important part of the experience of chronic widespread pain. Controllable (or uncontrollable) time can be identified in accounts relating to management of the body in exercise, carrying out household chores, carrying out self-care routines, family routines with regard to use of space; in short, those "mundane, repetitive and taken-for-granted aspects of everyday life" identified as being of considerable significance in the experiences of individuals [2]. The need to manipulate time and re-define it according to one's changing needs is important.

One of the limitations of this study is in the nature of the sample. All but one of the participants had stopped work due to ill health, which may have created a particular relationship with time. Future research could usefully focus on the experience of time in the context of being in paid employment and having chronic widespread pain. It would also be useful to explore whether people with chronic regional pain conditions experience time in the same way as people with chronic widespread pain. The strength of this study lies in the in-depth understanding it provides, through the combination of data generation methods, of the experience for people and their families of living with a condition such as chronic widespread pain. This understanding encompasses both a broader biographical perspective and a perspective that gives prominence to the routine and everyday. It also builds on our previous work in this area [7,8] on time and uncertainty in relation to people's perceptions of their futures with chronic widespread pain and to the disruptive effects on people's biographical lives.

For health care professionals, it may be helpful to recognise the different ways in which the uncertainty inherent in chronic widespread pain disrupts everyday experience of time. The experience of chronic widespread pain is one of constant adjustment and balancing between this uncertainty and other issues, including the need for routine, identity, enjoyment and fulfilment of roles within a family. An understanding of these phenomena will provide clinicians with a fuller understanding of how time plays an important role in patients' subjective experience of pain and their relationships with family members.

Understanding the issue of control and time in chronic widespread pain may enable health care professionals to support their patients in time management, for example, through adjusting pain relief, changing tasks and re-defining daily routines, providing advice on aids and adaptations and discussing formal and informal support. Moreover, it may also allow greater insight into the way in which patients incorporate treatments or management strategies within their daily lives, highlighting possible reasons for a lack of congruence between professionals' recommendations and patients' priorities, and allow realistic therapeutic timescales to be negotiated. In particular, it is likely that professionals will be better able to assist and advise patients regarding appropriate strategies and support to deal with the challenges of everyday function in the face of chronic pain.

## Competing interests

The author(s) declare that they have no competing interests.

## Authors' contributions

JCR, BNO and JS planned and designed the study. JCR carried out data collection. All authors were involved in analysis. All authors read and approved the final manuscript.

## Pre-publication history

The pre-publication history for this paper can be accessed here:


